# Attrition of methylnaltrexone treatment-emergent adverse events in patients with chronic noncancer pain and opioid-induced constipation: a post hoc pooled analysis of two clinical trials

**DOI:** 10.12688/f1000research.51073.2

**Published:** 2023-07-11

**Authors:** Neel Mehta, Neal E. Slatkin, Robert J. Israel, Nancy Stambler

**Affiliations:** 1 Weill Cornell School of Medicine, New York, NY, USA; 2 Salix Pharmaceuticals, Bridgewater, NJ, USA; 3 School of Medicine, University of California Riverside, Riverside, CA, USA; 4 Bausch Health US, LLC, Bridgewater, NJ, USA; 5 Progenics Pharmaceuticals, Inc., a subsidiary of Lantheus Holdings Inc., New York, NY, USA

**Keywords:** methylnaltrexone, opioid analgesic, constipation, chronic pain, adverse events

## Abstract

**Background:** Opioids prescribed for the management of chronic noncancer pain are associated with nausea, vomiting, and constipation. Methylnaltrexone, a peripherally acting µ-opioid receptor antagonist, has demonstrated robust efficacy and was well-tolerated in treating opioid-induced constipation without affecting central analgesia. Our objective was to assess changes in the frequency of treatment-emergent adverse events (TEAEs) after the first or second dose of methylnaltrexone or placebo.

**Methods:** This post hoc analysis pooled data from two randomized, placebo-controlled clinical trials assessing methylnaltrexone for opioid-induced constipation in the outpatient setting. Patients received subcutaneous methylnaltrexone (12 mg once daily or 12 mg once every other day), oral methylnaltrexone (150, 300, or 450 mg daily), or placebo. TEAEs, opioid withdrawal symptoms, pain intensity, and rescue-free bowel movements (RFBMs) within 4 hours of the first dose (i.e., RFBM responders) were assessed. Associations between TEAE frequencies and RFBM response were also evaluated.

**Results:** The analysis included 1263 adult patients with chronic noncancer pain. TEAE rates declined from treatment day 1 to 2 (methylnaltrexone: 16.2%–5.3%; placebo: 6.6%−5.4%). Among methylnaltrexone-treated patients, significantly greater proportions of RFBM responders versus nonresponders reported gastrointestinal TEAEs on day 1. No associations between RFBM response and the frequency of TEAEs were observed in the placebo group. No meaningful changes in opioid withdrawal symptoms or pain intensity were observed.

**Conclusions: **Early-onset TEAEs following methylnaltrexone treatment, particularly gastrointestinal TEAEs, are at least partially due to laxation. Methylnaltrexone treatment effectively relieves opioid-induced constipation without affecting the central analgesic effects of opioids.

## Introduction

Opioids, despite their potential drawbacks, remain an analgesic mainstay for patients with a number of chronic refractory pain conditions, including appropriate patients with chronic noncancer pain. The use of opioids, even over the short-term, may be associated with gastrointestinal side effects, such as nausea, abdominal pain, vomiting, and constipation. ^1^^–^^4^ Of these, constipation has been ranked by patients as the most bothersome, ^2^ and can have a demonstrably negative impact on quality of life. ^5^^,^^6^ Opioid-induced constipation (OIC) occurs in as many as 80% of patients treated with opioids, ^7^ frequently leading to dose reduction or discontinuation of therapy. ^1^^,^^2^^,^^4^^,^^8^^,^^9^ Moreover, whereas other gastrointestinal side effects associated with opioids tend to dissipate over time, OIC is generally not subject to the development of tolerance and, therefore, presents a patient management challenge requiring ongoing assessment, monitoring, and treatment. ^1^ Preventative measures and common constipation remedies, including lifestyle changes and over-the-counter or prescription laxatives, only provide limited relief from OIC. ^8^^,^^10^^,^^11^

Methylnaltrexone (Relistor ^®^, Salix Pharmaceuticals, a division of Bausch Health US, LLC, Bridgewater, NJ) is a peripherally acting μ-opioid receptor antagonist that reverses opioid-induced effects in the gastrointestinal tract, such as delayed gastric emptying and prolonged oral-cecal transit time. ^12^^,^^13^ Pain relief with opioid therapy, however, is maintained during methylnaltrexone treatment because the high polarity and low lipid solubility of the molecule inhibits its passage through the blood-brain barrier, thereby preserving centrally mediated opioid analgesia. ^14^^,^^15^ Methylnaltrexone is available in subcutaneous and oral formulations, both of which are approved for the treatment of OIC in adults with chronic noncancer pain. ^16^ Subcutaneous methylnaltrexone is also indicated for the treatment of OIC in patients with advanced illness or pain caused by active cancer. ^16^^–^^18^

In clinical trials, the majority of treatment-emergent adverse events (TEAEs) that occurred during methylnaltrexone treatment were gastrointestinal in nature (e.g., abdominal pain, diarrhea, nausea). ^15^^,^^19^^–^^21^ As many of these events are also common during laxation, it is plausible that gastrointestinal TEAEs reported in patients who received methylnaltrexone, most of whom had not had an adequate response to their laxative regimens before entering the studies, are of short duration and may be linked to successful relief of OIC. To test this hypothesis, the frequency of TEAEs after multiple doses of methylnaltrexone in two randomized, placebo-controlled clinical trials were evaluated. Relationships between TEAE frequency and methylnaltrexone efficacy, measured by opioid withdrawal symptom frequency, changes in pain intensity, and rescue-free bowel movements (RFBMs) within 4 hours of the first study drug dose, were also evaluated.

## Methods

### Study design

A post hoc analysis was performed using pooled data from two randomized, double-blind, placebo-controlled clinical trials that evaluated the efficacy and safety of subcutaneous or oral methylnaltrexone for the relief of OIC in patients with chronic, noncancer pain ( NCT00529087 [Study dates August 22, 2007 – November 25, 2008], NCT01186770 [Study dates September 1, 2010 – November 8, 2011). Study methodologies for both clinical trials have been previously published. ^15^^,^^19^^,^^20^ Briefly, the studies enrolled adult patients who had chronic noncancer pain for at least two months and OIC for at least 30 days. The presence of OIC was confirmed during screening and defined as fewer than three RFBMs (no laxative use within 24 hours prior to the bowel movement) per week on average and one or more of the following symptoms: hard or lumpy stools, straining during bowel movements, or a sensation of incomplete evacuation after bowel movements. Patients were required to have been receiving an opioid for at least one month, with a daily dose of at least 50-mg oral morphine equivalents for 14 days prior to screening. Patients with a history of clinically significant bowel or rectal disease, chronic constipation, unstable hepatic, renal, pulmonary, cardiovascular, ophthalmologic, neurologic, psychiatric or any other medical condition that might compromise the study or put the patient at risk were excluded from the studies. Each study was approved by independent ethics committees at each participating institution and was conducted in accordance with the International Conference on Harmonisation Guideline for Good Clinical Practice and the Declaration of Helsinki. All patients provided written informed consent.

Patients in the subcutaneous methylnaltrexone study were randomized 1:1:1 to receive treatment with methylnaltrexone 12 mg once daily, methylnaltrexone 12 mg every other day, or placebo for 4 weeks. Patients then entered an 8-week, open-label phase, during which methylnaltrexone was administered to all patients on an as-needed basis. Patients participating in the oral methylnaltrexone study were randomized 1:1:1:1 to receive treatment with methylnaltrexone 150 mg, 300 mg, 450 mg, or placebo once daily for 4 weeks, then as needed for an additional 8 weeks during an open-label study phase. In both studies, patients discontinued use of laxatives prior to study enrollment. Rescue laxative use (one dose of up to 3 or 4 bisacodyl tablets) was permitted if the patient had no bowel movements for three consecutive days. Rescue laxative use was limited to a single dose within a 24-hour period administered 4 hours or more after study drug administration.

### Assessments

Safety and tolerability on treatment days 1 and 2 were evaluated by TEAE rates and severity. Opioid withdrawal symptoms were measured by the patient and by the clinician using the Subjective Opiate Withdrawal Scale (SOWS) and the Objective Opiate Withdrawal Scale (OOWS), respectively. For the SOWS, patients rated their perceived severity of 19 opioid withdrawal symptoms on a scale from 0 (not at all) to 4 (extremely), with a total possible score of 76. The original SOWS scale has 16 questions ^22^; three questions were added for the purpose of this study to more accurately reflect withdrawal symptoms in a study population with OIC. The three additional statements regarding symptoms included: I have had trouble sleeping; My appetite has been poor; and I have had diarrhea. Additionally, the original SOWS statement of “I feel like shooting up now” was modified to “I have felt like taking more pain medication”. For the OOWS, clinicians assigned patients a score of 0 or 1 based on the absence or presence of 13 symptoms indicative of opioid withdrawal, with a total possible score of 13. In addition to SOWS and OOWS total scores, each scale was also evaluated without inclusion of cramping as a symptom, because cramping may also be associated with constipation and the process of laxation and, therefore, may confound the assessment of opioid withdrawal symptoms. ^14^ Evaluations of SOWS and OOWS were performed at 1 hour postdose on day 1 and at weeks 2 and 4 during the double-blind treatment phases of the studies. Maintenance of analgesia was assessed via a pain intensity score reflecting patients’ ratings of the intensity of their pain on a scale from 0 (no pain) to 10 (worst pain possible) at each study visit. ^15^ Efficacy was measured by the proportion of patients demonstrating a laxation response to treatment, defined for the purpose of this analysis as an RFBM within 4 hours of first study drug dose.

### Statistical analysis

The analysis population consisted of all randomized patients pooled from both included studies. Demographics and TEAEs were summarized using descriptive statistics. Between-group comparisons in RFBM responders were performed using chi-square tests. Associations between individual TEAEs and the occurrence of an RFBM within 4 hours of the first study drug dose were evaluated using Fisher’s exact test. Changes from baseline in SOWS and OOWS between groups were assessed by analysis of covariance, with treatment as the main effect and the baseline value as a covariate. Statistical calculations compared the all methylnaltrexone group versus placebo. All p-values reported for between-group comparisons used a nominal value of 0.05 to denote statistical significance. There were no corrections for multiplicity performed in these exploratory analyses. Statistical analyses were performed using SAS version 9.4 software.

## Results

A total of 1263 patients who received at least one dose of study medication were included in the pooled analysis: 900 had been randomized to methylnaltrexone (subcutaneous, n = 298; oral, n = 602) and 363 had been randomized to placebo. Patients in the subcutaneous methylnaltrexone treatment group were evenly divided between those who received 12 mg once daily (n = 150) and 12 mg every other day (n = 148). Among those in the oral methylnaltrexone treatment group, 201, 202, and 200 patients were randomized to treatment with methylnaltrexone 150 mg, 300 mg, and 450 mg once daily, respectively. Among all patients, 88% completed the double-blind phase. The discontinuation rate ranged from 10.0% to 18.8% depending on the methylnaltrexone dose and route of administration. The most common reasons for discontinuation were TEAEs, patient request, and protocol violations. Patients who discontinued due to TEAEs most commonly reported gastrointestinal complaints, such as abdominal pain, nausea, and vomiting ( Table 1).

**Table 1.** Patient disposition, reasons for discontinuation and discontinuation rates due to TEAEs.

Parameter	Placebo (n = 363)	Methylnaltrexone			Total (N = 1263)
		SC (n = 298)	Oral (n = 602)	All (n = 900)	
**Double-blind phase completed, n (%)**	326 (89.8)	242 (81.2)	543 (90.2)	785 (87.2)	1111 (88.0)
**Discontinued, n (%)**	37 (10.2)	56 (18.8)	60 (10.0)	116 (12.9)	153 (12.1)
TEAE	8 (2.2)	23 (7.7)	10 (1.7)	33 (3.7)	41 (3.2)
Failed to return/lost to follow-up	1 (0.3)	10 (3.4)	11 (1.8)	21 (2.3)	22 (1.7)
Protocol violation	13 (3.6)	15 (5.0)	5 (0.8)	20 (2.2)	33 (2.6)
Patient request	9 (2.5)	6 (2.0)	22 (3.7)	28 (3.1)	37 (2.9)
Ineligibility	0	0	2 (0.3)	2 (0.2)	2 (0.2)
Insufficient response	3 (0.8)	0	9 (1.5)	9 (1.0)	12 (1.0)
Other	3 (0.8)	2 (0.7)	1 (0.2)	3 (0.3)	6 (0.5)
**Discontinuations due to TEAEs > 2% of patients, n (%)**					
Abdominal pain	0	8 (2.7)	2 (0.3)	10 (1.1)	10 (0.8)
Nausea	0	6 (2.0)	0	6 (0.7)	6 (0.5)
Vomiting	1 (0.3)	4 (1.3)	1 (0.2)	5 (0.6)	6 (0.5)
Hyperhidrosis	0	4 (1.3)	1 (0.2)	5 (0.6)	5 (0.4)

SC = subcutaneous; TEAEs = treatment-emergent adverse events.

Demographic and baseline characteristics were generally well balanced among treatment groups ( *P*>0.05 for all methylnaltrexone vs placebo, Table 2). Patients in the oral methylnaltrexone treatment group reported modestly lower rates of baseline laxative use and a slightly greater mean number of RFBMs per week compared with patients who received subcutaneous methylnaltrexone. Baseline median daily morphine-equivalent doses and baseline mean pain scores were comparable among treatment groups.

**Table 2.** Patient demographics and baseline characteristics.

Parameter	Placebo (n = 363)	Methylnaltrexone			Total (N = 1263)
		SC (n = 298)	Oral (n = 602)	All (n = 900)	
Mean age, years (range)	51.3 (23, 83)	48.3 (23, 78)	51.3 (18, 82)	50.3 (18, 82)	50.6 (18, 83)
Gender, n (%)					
Men	134 (36.9)	120 (40.3)	227 (37.7)	347 (38.6)	481 (38.1)
Women	229 (63.1)	178 (59.7)	375 (62.3)	553 (61.4)	782 (61.9)
Race, n (%)					
White	307 (84.6)	272 (91.3)	494 (82.1)	766 (85.1)	1073 (85.0)
Black or African American	42 (11.6)	17 (5.7)	93 (15.4)	110 (12.2)	152 (12.0)
Other	14 (3.9)	9 (3.0)	15 (2.5)	24 (2.7)	38 (3.0)
Median baseline MED, mg/day (range)	145.3 (13.6, 1287)	160.0 (7.1, 1334)	151.0 (27.0, 2289)	152.5 (7.1, 2289)	150.0 (7.1, 2289)
Mean number of laxatives used (SD)	0.4 (0.6)	0.9 (0.5)	0.1 (0.4)	0.4 (0.5)	0.4 (0.6)
Mean RFBMs per week (SD)	1.3 (1.0)	1.0 (0.8)	1.4 (0.9)	1.3 (0.9)	1.3 (0.9)
Mean pain score (SD)	6.2 (1.9)	6.2 (1.9)	6.4 (1.9)	6.3 (1.9)	6.3 (1.9)

MED = morphine equivalent dose; RFBM = rescue-free bowel movements; SC = subcutaneous; SD = standard deviation.

### Adverse events

The numbers of patients who experienced at least one TEAE decreased from day 1 to day 2 of treatment among all treatment groups with the greatest decrease occurring in the subcutaneous methylnaltrexone treatment group ( Table 3). On treatment day 2, the overall incidence of TEAEs among all patients who received methylnaltrexone was similar to that of placebo.

**Table 3.** TEAEs ^a^ occurring on treatment day 1 and day 2.

TEAE	Patients, n (%)									
	Placebo		SC Methylnaltrexone QD		SC Methylnaltrexone QOD ^b^		Oral Methylnaltrexone		All Methylnaltrexone	
	Day 1 (n = 363)	Day 2 (n = 354)	Day 1 (n = 150)	Day 2 (n = 145)	Day 1 (n = 148)	Day 2 (n = 138)	Day 1 (n = 602)	Day 2 (n = 571)	Day 1 (n = 900)	Day 2 (n = 854)
Patients with at least 1 TEAE	24 (6.6)	19 (5.4)	33 (22.0)	10 (6.9)	31 (20.9)	8 (5.8)	82 (13.6)	27 (4.7)	146 (16.2)	45 (5.3)
Abdominal pain	3 (0.8)	4 (1.1)	17 (11.3)	7 (4.8)	11 (7.4)	1 (0.7)	24 (4.0)	6 (1.1)	52 (5.8)	14 (1.6)
Nausea	3 (0.8)	3 (0.8)	8 (5.3)	4 (2.8)	11 (7.4)	0	11 (1.8)	4 (0.7)	30 (3.3)	8 (0.9)
Hyperhidrosis	1 (0.3)	1 (0.3)	8 (5.3)	1 (0.7)	7 (4.7)	1 (0.7)	5 (0.8)	1 (0.2)	20 (2.2)	3 (0.4)
Diarrhea	0	0	3 (2.0)	1 (0.7)	8 (5.4)	2 (1.4)	3 (0.5)	1 (0.2)	14 (1.6)	4 (0.5)
Abdominal pain, upper	3 (0.8)	1 (0.3)	1 (0.7)	0	6 (4.1)	1 (0.7)	4 (0.7)	1 (0.2)	11 (1.2)	2 (0.2)
Vomiting	0	1 (0.3)	0	0	7 (4.7)	1 (0.7)	3 (0.5)	1 (0.2)	10 (1.1)	2 (0.2)
Hot flush	3 (0.8)	1 (0.3)	3 (2.0)	0	3 (2.0)	2 (1.4)	1 (0.2)	0	7 (0.8)	2 (0.2)

QD = daily; QOD = every other day; SC = subcutaneous; TEAEs = treatment-emergent adverse events.

^a^ Reported by ≥2% of patients in any treatment group.

^b^ Treatment day 2 occurred on study day 3 for patients who received SC methlynaltrexone every other day.

TEAEs reported on days 1 and 2 of treatment were predominantly gastrointestinal (e.g., abdominal pain, nausea, diarrhea, upper abdominal pain, and vomiting) ( Table 3). All TEAEs were reported by fewer patients on treatment day 2 compared with day 1 among patients who received methylnaltrexone. Abdominal pain was the most common TEAE reported on treatment day 1 among patients who received methylnaltrexone or placebo. Among the patients treated with methylnaltrexone who experienced abdominal pain on day 1, the majority (83%, n = 43/52) reported mild-moderate abdominal pain and 17% (n = 9/52) reported severe abdominal pain. All patients in the placebo group who reported abdominal pain on day 1 experienced mild-moderate pain (100%, n = 3/3). Abdominal pain on day 1 led to treatment discontinuation in 0.4% (n = 4) of methylnaltrexone-treated patients and in none of the patients who received placebo.

On treatment day 2, the frequency of abdominal pain had decreased among patients treated with methylnaltrexone, whereas the frequency among patients who received placebo was unchanged. Among patients reporting abdominal pain on treatment day 2, pain severity was characterized as mild-moderate in most of the patients treated with methylnaltrexone (87.5%, n = 14/16) and in all of the patients who received placebo (100%, n = 4/4). Two methylnaltrexone-treated patients (12.5%, n = 2/16) reported severe abdominal pain on treatment day 2. Abdominal pain on treatment day 2 led to treatment discontinuation in 0.2% (n = 2) of methylnaltrexone-treated patients; none discontinued treatment in the placebo group due to abdominal pain on treatment day 2. Hyperhidrosis and nausea frequency also markedly decreased from day 1 to day 2 of treatment in methylnaltrexone-treated patients. The frequency of TEAEs reported after the second dose of methylnaltrexone treatment were comparable to or less than those reported after the second dose of placebo ( Table 4).

**Table 4.** TEAEs ^a^ occurring after treatment day 2.

TEAE	Patients, n (%)			
	Placebo (n = 363)	SC Methylnaltrexone (n = 298)	Oral Methylnaltrexone (n = 602)	All Methylnaltrexone (n = 900)
Patients with at least 1 TEAE	203 (55.9)	152 (51.0)	309 (51.3)	461 (51.2)
Abdominal pain	28 (7.7)	28 (9.4)	21 (3.5)	49 (5.4)
Nausea	28 (7.7)	16 (5.4)	25 (4.2)	41 (4.6)
Diarrhea	19 (5.2)	18 (6.0)	32 (5.3)	50 (5.6)
Vomiting	18 (5.0)	5 (1.7)	12 (2.0)	17 (1.9)
Urinary tract infection	15 (4.1)	12 (4.0)	21 (3.5)	33 (3.7)
Upper respiratory tract infection	13 (3.6)	3 (1.0)	24 (4.0)	27 (3.0)
Flatulence	13 (3.6)	7 (2.3)	17 (2.8)	24 (2.7)
Back pain	12 (3.3)	8 (2.7)	21 (3.5)	29 (3.2)
Headache	12 (3.3)	12 (4.0)	15 (2.5)	27 (3.0)
Abdominal pain, upper	9 (2.5)	5 (1.7)	12 (2.0)	17 (1.9)
Influenza	8 (2.2)	3 (1.0)	12 (2.0)	15 (1.7)
Anxiety	7 (1.9)	1 (0.3)	16 (2.7)	17 (1.9)
Dizziness	3 (0.8)	7 (2.3)	5 (0.8)	12 (1.3)

SC = subcutaneous; TEAEs = treatment-emergent adverse events.

^a^ Reported by ≥2% of patients in any treatment group.

### Relief of OIC

The proportion of patients who experienced an RFBM within 4 hours after the first dose of study treatment (i.e., RFBM responders) was significantly greater among all patients who received methylnaltrexone (25.1%, n = 226/900) compared with placebo (8.8%, n = 32/363; *P* < 0.0001). In addition, more patients treated with subcutaneous versus oral methylnaltrexone were RFBM responders (34.2%, n = 102/298 and 20.6%, n = 124/602, respectively).

### Association between TEAE frequency and RFBM response

Associations between RFBM response and the occurrence of TEAEs on day 1 were evaluated. Among all methylnaltrexone-treated patients, abdominal pain on day 1 was reported by a significantly greater proportion of RFBM responders compared with nonresponders ( Table 5). Similarly, significantly greater proportions of RFBM responders versus nonresponders reported upper abdominal pain, diarrhea, and nausea on day 1 among all methylnaltrexone-treated patients. No statistically significant associations between the frequency of TEAEs and RFBM response were observed among patients who received placebo. Among the patients who received subcutaneous methylnaltrexone 12 mg daily or oral methylnaltrexone 450 mg daily (the doses currently approved by the US Food and Drug Administration for the treatment of OIC), greater proportions of patients who were responders (13.7%, n = 14/102 and 10.6%, n = 5/47, respectively) than nonresponders (7.7%, n = 15/196 and 5.2%, n = 8/153, respectively) reported abdominal pain on day 1.

**Table 5.** TEAEs ^a^ after the first dose of study drug and RFBM within 4 hours of dosing.

	Placebo (n = 363)			Methylnaltrexone (n = 900)		
	No RFBM within 4 hours of 1 ^st^ dose (n = 331)	RFBM within 4 hours of 1 ^st^ dose (n = 32)	*P*-Value	No RFBM within 4 hours of 1 ^st^ dose (n = 674)	RFBM within 4 hours of 1 ^st^ dose (n = 226)	*P*-Value
Abdominal pain, n (%)	3 (0.9)	0	NS	28 (4.2)	24 (10.6)	0.0008
Abdominal pain, upper, n (%)	3 (0.9)	0	NS	4 (0.6)	7 (3.1)	0.0074
Diarrhea, n (%)	0	0	NS	6 (0.9)	8 (3.5)	0.0101
Nausea, n (%)	3 (0.9)	0	NS	16 (2.4)	14 (6.2)	0.0092
Hyperhidrosis, n (%)	1 (0.3)	0	NS	13 (1.9)	7 (3.1)	NS

NS = not significant, *P*>0.05; RFBM = rescue-free bowel movement; TEAEs = treatment-emergent adverse events.

^a^ Reported by ≥2% of patients in any treatment group.

### Opioid withdrawal and maintenance of analgesia

**Figure 1.**
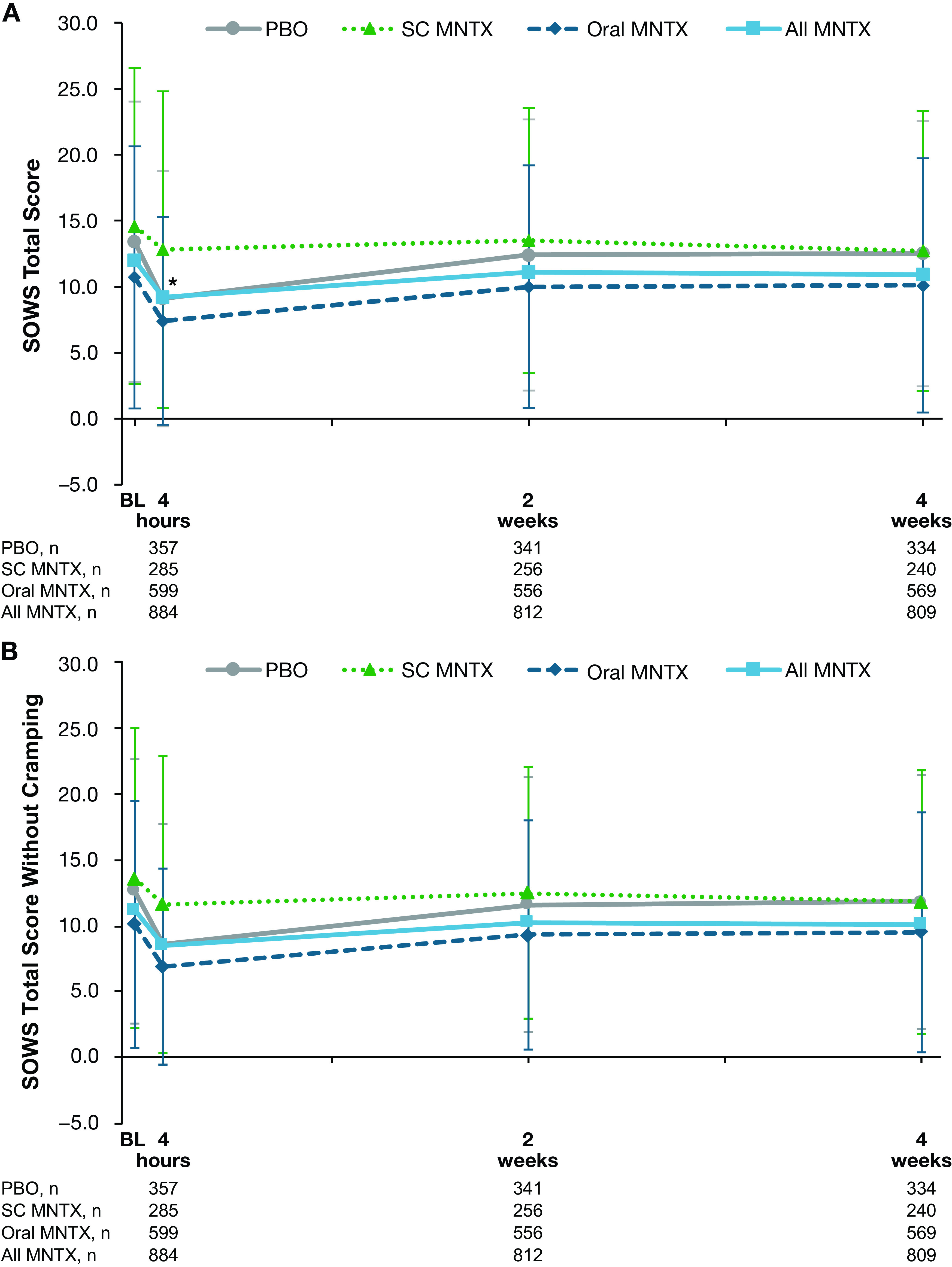
Effect of study treatment on SOWS (A) total score and (B) total score without cramping. Data are presented as means ± standard deviations.* *P* < 0.05 for the comparison of change from baseline in least-squares mean values in the all methylnaltrexone vs placebo treatment groups. BL = baseline; MNTX = methylnaltrexone; PBO = placebo; SC = subcutaneous; SOWS = Subjective Opioid Withdrawal Scale.

In all treatment groups, slight decreases in SOWS total scores were observed between baseline and the day 1 postdose assessment, with the least decline occurring in the subcutaneous methylnaltrexone treatment group ( Figure 1A). The difference in decrease from baseline in SOWS total scores between treatment groups was statistically significant for the comparison of the combined methylnaltrexone treatment group versus placebo at day 1 (least-squares means, −3.6 and −2.6, respectively; *P* = 0.01), but was not statistically significant at weeks 2 or 4. Similar results were observed for SOWS total scores without cramping ( Figure 1B).

**Figure 2.**
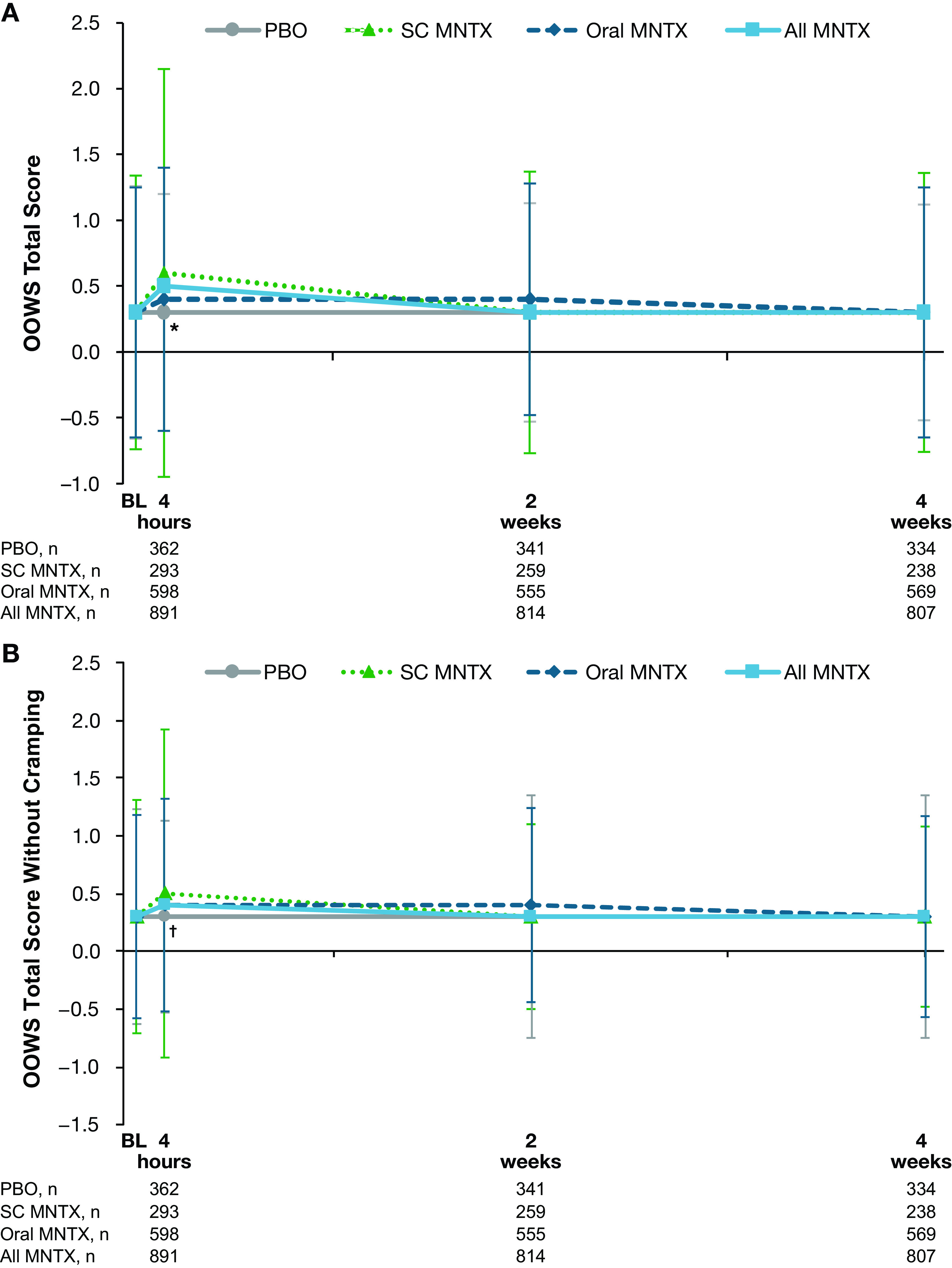
Effect of study treatment on OOWS (A) total score and (B) total score without cramping. Data are presented as means ± standard deviations. * *P =* 0.001; ^†^ *P* < 0.05; for the comparison of change from baseline in least-squares mean values in the all methylnaltrexone vs placebo treatment groups. BL = baseline; MNTX = methylnaltrexone; OOWS = Objective Opioid Withdrawal Scale; PBO = placebo; SC = subcutaneous.

The OOWS total scores increased slightly from baseline to the day 1 postdose assessment in all methylnaltrexone treatment groups, whereas the placebo score remained unchanged ( Figure 2A). The difference in changes from baseline values between the combined methylnaltrexone treatment group and placebo was statistically significant at day 1 (least-squares means, 0.13 and −0.02, respectively; *P*=0.001), but not at weeks 2 or 4. When cramping was omitted from the OOWS total score, the observed increases from baseline score in the methylnaltrexone treatment group lessened but remained significantly different from placebo at day 1 ( Figure 2B).

**Figure 3.**
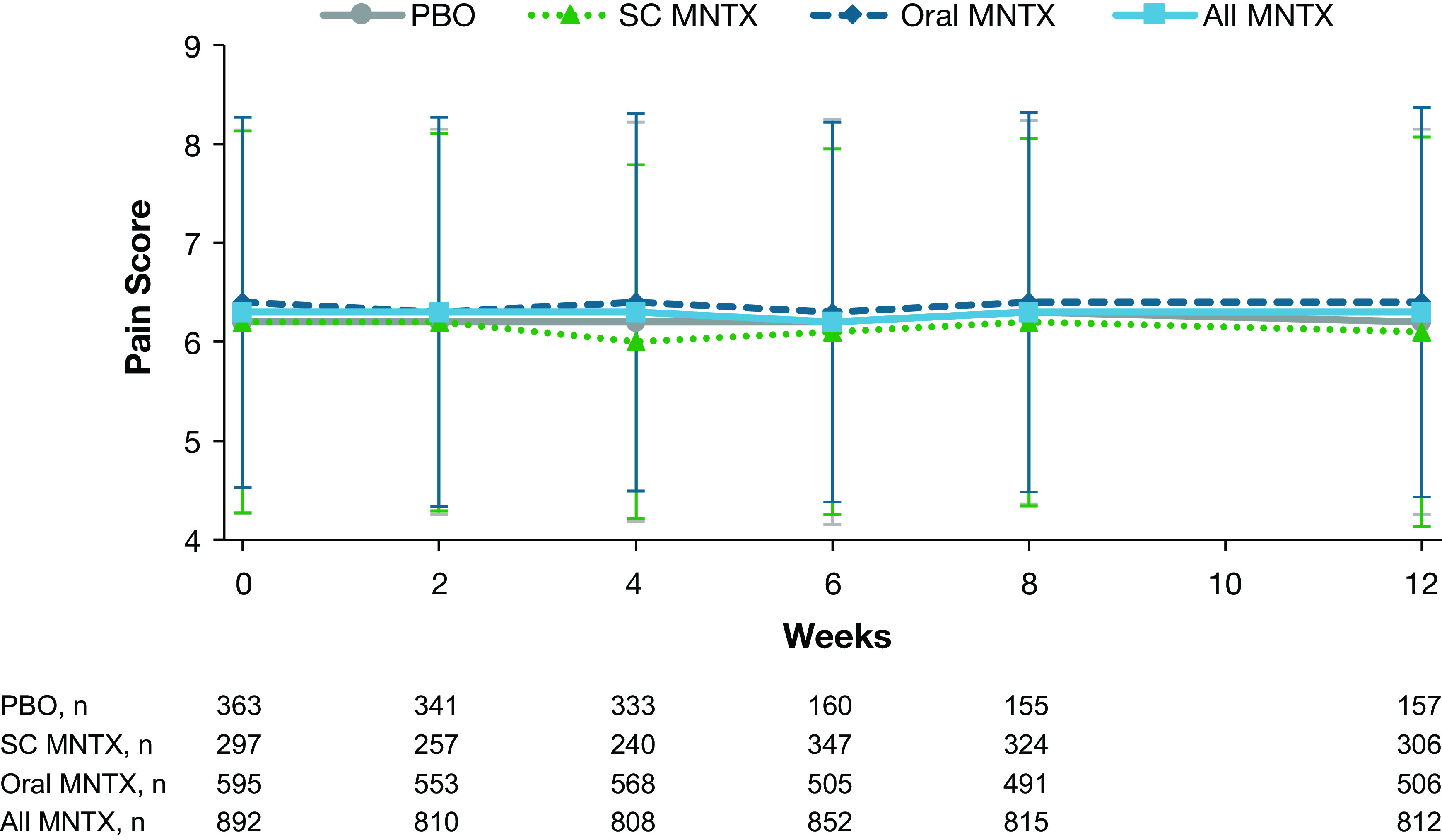
Pain scores during treatment with methylnaltrexone or placebo. Data are presented as means ± standard deviations. MNTX = methylnaltrexone; PBO = placebo; SC = subcutaneous.

Pain intensity scores did not change significantly from baseline for any treatment group throughout the study ( Figure 3). Least-squares mean changes in pain intensity score ranged from −0.02 to −0.12.

## Discussion

In this pooled analysis of patients with chronic, noncancer pain and OIC, rates of TEAEs decreased considerably between the first and second dosing days following treatment with methylnaltrexone and were comparable to placebo after the second dose. Abdominal pain, nausea, hyperhidrosis, and diarrhea were the most frequently reported TEAEs in the methylnaltrexone treatment group at day 1 and also demonstrated the most pronounced decreases in frequency after the second dose. The presence of abdominal pain was predominantly reported as mild or moderate in intensity, and very few patients discontinued due to abdominal pain. An association was detected between the presence of the gastrointestinal symptoms of abdominal pain, upper abdominal pain, nausea, and diarrhea on day 1 and the occurrence of an RFBM within 4 hours of the first methylnaltrexone dose. Together, these data demonstrate the rapid attrition of early-onset TEAEs occurring with methylnaltrexone treatment for OIC, and suggest that gastrointestinal symptoms may be due, in part, to resumption of bowel function/constipation relief.

A similar decrease in abdominal pain frequency from the first to second dose of methylnaltrexone was reported in a prior post hoc analysis using data from two randomized, placebo-controlled clinical trials of subcutaneous methylnaltrexone in patients with advanced illness whose laxative therapy response was insufficient (N=288). ^23^ In that study, abdominal pain was reported by 23% of patients following the first methylnaltrexone dose, by 13% of patients following the second dose, and by less than 10% of patients following the fifth dose, a rate similar to the frequency of abdominal pain reported by the placebo group (9.8%). The investigators also observed a relationship between abdominal pain and laxation response. In total, 80% of patients in the methylnaltrexone treatment group who experienced abdominal pain on study day 1 had an RFBM within 4 hours of the first study drug dose, whereas 47.2% of patients without abdominal pain on day 1 demonstrated an RFBM response. These data support the hypothesis that, in patients with OIC despite ongoing laxative use, the process of being rapidly induced to a bowel movement is initially accompanied by abdominal pain with the first dose, but once laxation has occurred, subsequent doses are generally not accompanied by such pain.

In the current study, safety and efficacy assessments were generally comparable between the subcutaneous and oral methylnaltrexone formulations, although the frequency of TEAEs after the first dose of study drug and the decrease in TEAE rates from day 1 to treatment day 2 were greater among patients treated with subcutaneous methylnaltrexone. There are intrinsic and study-design related factors that could contribute to this observed difference between formulations. First, patients who received subcutaneous methylnaltrexone had a greater response rate (i.e., RFBM within 4 hours of first study drug dose) compared with patients who received oral methylnaltrexone. It has been postulated that the greater initial response rate is due to a faster onset of effect with the subcutaneous formulation, which, unlike the oral formulation, does not require time for absorption. ^20^ If the hypothesis that the frequencies of the observed gastrointestinal symptoms are partially due to OIC relief is correct, then a greater rate of RFBM response within 4 hours would naturally be linked to a greater frequency of early-onset gastrointestinal TEAEs consistent with laxation, such as abdominal pain and cramping. Second, the study of oral methylnaltrexone investigated three doses, the lower doses being one third (150 mg) and two thirds (300 mg) of the recommended methylnaltrexone dose (450 mg). As oral methylnaltrexone efficacy has been shown to be dose dependent, ^20^ the inclusion of lower doses in this analysis may have influenced RFBM response and the frequency of any associated TEAEs. However, when the patients receiving the methylnaltrexone doses that are approved for OIC treatment (subcutaneous methylnaltrexone 12 mg/day or oral methylnaltrexone 450 mg/day) were analyzed separately, more patients who were responders than nonresponders reported having abdominal pain on day 1, indicating that treatment with the approved doses may have an effect on efficacy. In addition, when discontinuation rates were assessed for each dose and regimen, overall discontinuation rates and discontinuation rates due to TEAEs were consistent between the approved doses (subcutaneous methylnaltrexone 12 mg/day and oral methylnaltrexone 450 mg/day) and the other studied doses (subcutaneous methylnaltrexone 12 mg every other day and oral methylnaltrexone 150 or 300 mg/day), further supporting the safety profile of the approved doses.

Pooled data from the two included clinical trials indicate that methylnaltrexone does not induce symptoms of opioid withdrawal. Scores for SOWS and OOWS showed slight changes after the initial study drug dose but returned to baseline levels by the subsequent assessment and were stable thereafter. Early changes in SOWS and OOWS scores could be partially attributable to changes in gastrointestinal TEAE frequency, as several items in both assessments address gastrointestinal symptoms. ^22^ Interestingly, the initial decrease from baseline in SOWS was greatest in the placebo group. The clinical significance of a decrease from baseline in SOWS score is not clear, as it insinuates that a patient had symptoms of opioid withdrawal prior to receiving methylnaltrexone that were lessened by treatment. As pain intensity scores were consistent throughout the 12-week study durations and compromised analgesia typically precedes symptoms of opioid withdrawal in patients with chronic pain, ^24^^,^^25^ the clinical significance of the SOWS and OOWS score changes observed in this study are even more questionable. Further studies evaluating and validating the use of the OOWS and SOWS in patients taking opioids for chronic nonmalignant pain without an opioid addiction are needed. However, data from this analysis affirm that neither subcutaneous nor oral methylnaltrexone negatively influences opioid-mediated analgesia. Lack of opioid withdrawal symptoms and maintenance of analgesia are consistent with methylnaltrexone’s pharmacologic profile and lack of effect on centrally mediated analgesia. ^14^^,^^15^

There are limitations that need to be considered when interpreting the findings from this analysis. The designs of the two studies were similar, but not identical, which adds potential confounding factors to the analysis. As mentioned above, the oral methylnaltrexone formulation needs time to be absorbed prior to producing any effects not required by the subcutaneous formulation, which could influence the timing of treatment effects. In addition, individual subcutaneous and oral dose groups were combined for the purposes of this analysis, and oral methylnaltrexone efficacy has been shown to be dose dependent. ^20^ However, in the individual published studies, the total numbers of TEAEs did not vary appreciably among dose groups ^19^^,^^20^ thus any influence on the TEAE attrition assessment is likely to be minimal. The observation period of both studies was limited: opioid withdrawal symptoms were only reported during the 4-week, double-blind treatment period, and pain intensity over 12 weeks. Longer-term data regarding the impact of methylnaltrexone on these parameters is available from a 48-week, open-label study, in which no indications of opioid withdrawal, loss of opioid-mediated analgesia, or alteration in median morphine equivalent dose were observed during methylnaltrexone treatment. ^21^

## Conclusion

The attrition of TEAEs after the first dose of methylnaltrexone and the association between gastrointestinal TEAEs and laxation response support the hypothesis that early-onset TEAEs experienced with methylnaltrexone treatment, particularly gastrointestinal TEAEs, are at least partially due to laxation. Treatment with methylnaltrexone was additionally shown to relieve OIC without inducing withdrawal symptoms or compromising analgesia. For patients with chronic pain and OIC, methylnaltrexone offers a well-tolerated and effective treatment option for constipation relief.

## Data availability

### Underlying data

Vivli: Attrition of TEAEs: Post Hoc Pooled Analysis, https://doi.org/10.25934/00007291. ^26^

Per the study sponsor’s policy, the datasets generated and/or analyzed for this study are not publicly available. Access to the data is provided to bona fide researchers subject upon submission of a research proposal and signing a Data Use Agreement. Interested researchers can request access to the data at the DOI by creating a free Vivli account and using the ‘Prepare to Request Vivli Study’ button on the ‘Administrative Details’ tab.
