# Supplementary material for: Attrition of methylnaltrexone treatment-emergent adverse events in patients with chronic noncancer pain and opioid-induced constipation: a post hoc pooled analysis of two clinical trials
Source: F1000Res. 2023 Jul 11;10:891. Originally published 2021 Sep 6. [Version 2] doi: 10.12688/f1000research.51073.2 (PMC8485099; doi:10.12688/f1000research.51073.2)
Supplement: Peer review history

# Peer review: Reviewer response for version 1

**DOI:** 10.5256/f1000research.54184.r94039

**Published:** 2021-09-30

**Authors:** Nebojsa Nick Knezevic

**Affiliations:** 1 Department of Anesthesiology, Advocate Illinois Masonic Medical Center, Chicago, IL, USA; 2 University of Illinois, Chicago, IL, USA

This post hoc analysis pools the results from two randomized, placebo-controlled clinical trials (n=1263) to examine the effect of subcutaneous or oral methylnaltrexone on adverse events in patients with opioid-induced constipation. All patients had chronic, non-cancer pain. The authors conclude that early-onset adverse events experienced in the methylnaltrexone group may be the result of laxation related to rescue-free bowel movements (RFBM).

- In the title and several parts of the article (regarding Table 3 in particular), the authors use the term “treatment-emergent adverse events”, yet throughout most of the article they simply use “adverse events.” How was this distinction made?
- Few statistical analyses are presented in the paper. When describing the results of the Tables, the authors often use terms such as “generally well-balanced”, “comparable”, “modestly lower” and “slightly greater”. When describing discontinuation rates (Table 1), patient baseline characteristics (Table 2), and adverse events (Tables 3 and 4), it would be relevant to use statistical significance to characterize similarities and differences between groups.

Is the work clearly and accurately presented and does it cite the current literature?

Yes

If applicable, is the statistical analysis and its interpretation appropriate?

Yes

Are all the source data underlying the results available to ensure full reproducibility?

Partly

Is the study design appropriate and is the work technically sound?

Yes

Are the conclusions drawn adequately supported by the results?

Yes

Are sufficient details of methods and analysis provided to allow replication by others?

Yes

Reviewer Expertise:

chronic pain management

I confirm that I have read this submission and believe that I have an appropriate level of expertise to confirm that it is of an acceptable scientific standard.

# Response

**Published:** 2023-04-06

**Authors:** Neel Mehta

**Affiliations:** Weill Cornell Medical Center, USA

There was no distinction in use of “treatment-emergent adverse events” and “adverse events”, this was more for ease of reading. We have revised all instances to “TEAE”.

 We have added more detail in the baseline demographics section that clarifies P>0.05 for methylnaltrexone vs placebo for all baseline parameters. We did not perform any other statistical analyses.

# Peer review: Reviewer response for version 1

**DOI:** 10.5256/f1000research.54184.r94041

**Published:** 2021-09-28

**Authors:** Mayank Gupta

**Affiliations:** 1 Department of Anaesthesiology and Critical Care, All India Institute of Medical Sciences, Bathinda, Punjab, India

Thank you for giving me the opportunity to review the manuscript.

 This post hoc analysis study used data from two RCTs evaluating methylnaltrexone for opioid-induced constipation. The study aimed to find an association (if any) between the methylnaltrexone induced adverse effects (AEs) and its clinical effect i.e. Rescue free bowel movements. The results of the study demonstrate a statistically significant association between the AEs and the intended clinical effect i.e. laxation.

 However, the authors missed citing key recent work done by Chamberlain BH *et al.* ^1^ and Nelson KK *et al.* ^2^ who have evaluated the efficacy of methylnaltrexone for opioid-induced constipation. Rather than just citing package insert (reference number 16), it would be prudent to cite references like above when the authors mention the indications of Subcutaneous methylnaltrexone for opioid-induced constipation in advanced illnesses in the introduction part.  

 Overall, a good article using a robust methodology for an outcome of clinical significance.

Is the work clearly and accurately presented and does it cite the current literature?

Partly

If applicable, is the statistical analysis and its interpretation appropriate?

Yes

Are all the source data underlying the results available to ensure full reproducibility?

Yes

Is the study design appropriate and is the work technically sound?

Yes

Are the conclusions drawn adequately supported by the results?

Yes

Are sufficient details of methods and analysis provided to allow replication by others?

Yes

Reviewer Expertise:

Cancer pain, palliative care

I confirm that I have read this submission and believe that I have an appropriate level of expertise to confirm that it is of an acceptable scientific standard.

## References

[1] : Subcutaneous methylnaltrexone for opioid-induced constipation in advanced-illness patients with or without active cancer. *Pain Manag* .2020;10(2) : 10.2217/pmt-2019-0045 73-84 10.2217/pmt-2019-0045 31951150

[2] : Methylnaltrexone is safe in cancer patients with peritoneal carcinomatosis. *Scientific Reports* .2019;9(1) : 10.1038/s41598-019-44864-2 10.1038/s41598-019-44864-2 31270339PMC6610621

# Response

**Published:** 2023-04-06

**Authors:** Neel Mehta

**Affiliations:** Weill Cornell Medical Center, USA

We have added these references to the introduction.
